# Applications of silver nanoparticles synthesized from *Pichia kudriavzevii* bioflocculant isolated from Kombucha tea SCOBY

**DOI:** 10.1016/j.biotno.2025.02.003

**Published:** 2025-02-21

**Authors:** Phakamani H. Tsilo, Albertus K. Basson, Zuzingcebo G. Ntombela, Nkosinathi G. Dlamini, Rajasekhar V.S.R. Pullabhotla

**Affiliations:** a Department of Biochemistry and Microbiology, Faculty of Science, Agriculture and Engineering, University of Zululand, P/Bag X1001, KwaDlangezwa, 3886, South Africa; b Department of Chemistry, Faculty of Science, Agriculture and Engineering, University of Zululand, P/Bag X1001, KwaDlangezwa, 3886, South Africa; c Writing Centre: Teaching and Learning Centre, University of Zululand, P/Bag X1001, KwaDlangezwa, 3886, South Africa

**Keywords:** Silver nanoparticles, Removal efficiency, Kombucha, Bioflocculant, Antimicrobial, Wastewater treatment, Pollutants

## Abstract

Studying the utilization of natural products in the biosynthesis of silver nanoparticles (AgNPs) recently appears to be a fascinating area of research within nanotechnology. These nanoparticles exhibit biocompatibility and inherent stability, making them highly suitable for various industrial applications. The utilization of bioflocculant-synthesized Ag nanoparticles was investigated in this study for the purpose of eliminating diverse pollutants and dyes from wastewater and solutions. In this study, Ag nanoparticles were successfully synthesized through a green method utilizing a bioflocculant derived from *Pichia kudriavzevii* isolated from Kombucha tea SCOBY as a stabilizing agent. The resulting nanoparticles were then evaluated for their flocculation and antimicrobial properties. Different characterization techniques including SEM, EDX, FT-IR, TGA, and TEM were investigated from the synthesized nanoparticles. Furthermore, the cytotoxicity of the Ag nanoparticles was assessed on human embryonic kidney (HEK 293) cells. The EDX analysis showed elemental Ag constituted 61.93 wt% of the prepared AgNPs. SEM revealed particles with average size of 15.8 nm and were spherical in shape. Thermo-gravimetric analysis (TGA) demonstrated that AgNPs exhibited enhanced thermal stability, retaining over 85 % of their mass at elevated temperatures. In a concentration-dependent manner, the spherical biosynthesized nanoparticles exhibited notable cytotoxic effects on HEK 293 cell lines with over 68 % cell viability at 25 mg/mL concentration. The biosynthesized Ag nanoparticles displayed robust antimicrobial efficacy against both Gram-positive and Gram-negative pathogenic bacteria, though Gram-negative were more susceptible with MIC of 3.125 mg/mL concentration. The nanoparticles showcased a dye removal efficiency exceeding 78 % for all the tested dyes with highest removal efficiency of 96 % for methylene blue at a dosage concentration of 0.2 mg/mL of AgNPs. The Ag nanoparticles exhibited exceptional efficiencies in removing a wide range of pollutants present in wastewater. Compared to traditional flocculants, the biosynthesized Ag nanoparticles demonstrated significant potential in effectively removing both biological oxygen demand (BOD) (92 % removal efficiency) and chemical oxygen demand (COD) (86 % removal efficiency). Thus, the biosynthesized Ag nanoparticles show great potential as a substitute for chemical flocculants in the treatment of industrial wastewater, offering im-proved purification capabilities.

## Highlights

• Silver nanoparticles were biosynthesized using a bioflocculant from Pichia kudriavzevii isolated from Kombucha tea SCOBY.
• A removal efficiency of above 78% was observed for all tested dyes with methylene blue showing 96% removal rate.
• Biosynthesized AgNPs removed 92% BOD and 86% COD from industrial wastewater, outperforming traditional flocculants.
• The AgNPs showed cytotoxic effects on HEK 293 cells, with over 68 % cell viability at 25 μg/L concentration.
• Biosynthesized AgNPs offer a sustainable, efficient alternative to chemical flocculants, enhancing wastewater purification.

## 1 Introduction

The provision of safe drinking water is a significant issue for the well-being of humans and animals alike. In numerous countries, water scarcity is closely linked to climate fluctuations, industrialization, and population expansion, particularly in developing nations.^1^ Various techniques are employed in the purification of water, including flotation/filtration, sedimentation, heating/boiling, and salting-in techniques.^2^ Chemical-based water purification has been regarded as the preferred choice for water treatment due to its exceptional efficacy and cost-effectiveness. Nonetheless, the use of commercial chemicals in water purification poses negative consequences, as they are ecologically unfriendly and can be harmful to human health.^3^

Conversely, bioflocculants have emerged as a focal point in scientific and biotechnological research due to their exceptional properties.^4^ Bioflocculants are secondary metabolites released by microorganisms, encompassing algae, fungi, and bacteria as they progress through their growth stages.^5^ Bioflocculants are macromolecules, including nucleic acids, proteins, glycoproteins, and carbohydrates, which are formed as by-products of microbial growth, substrate metabolism, and cell lysis.^6^ Bioflocculants possesses distinctive attributes such as their eco-friendly nature, biodegradability, and absence of secondary pollution during their utilization. Bioflocculants face challenges in terms of high production costs, low production yields, and lower efficiency when compared to traditional flocculants. These limitations currently render bioflocculants less commercially feasible in industrial applications.^7^

Nanotechnology has been heralded as the imminent industrial revolution of the century due to its rapid advancement and the potential to establish a foundation for integrated technological and biotechnological innovations.^8^ This method has found applications in diverse industrial and academic disciplines, including medicine, physics, chemistry, and biology.^9^ Given their superior biological and physiochemical properties compared to bulk materials, nanoparticles hold immense potential across a wide range of scientific domains.^10^ Nanoparticles are substances characterized by their size ranging from 1 to 100 nm to at least one dimension, exhibiting altered chemical and physical properties.^11^^,^^12^ A variety of manufacturing methods have been documented for nanoparticles, encompassing both chemical and physical technologies. Nevertheless, both techniques suffer from certain drawbacks, including the need for high-energy input and utilization of potentially toxic reagents or chemicals.^13^ To address these limitations, metallic nanoparticles have been produced through biological approaches and harnessed for wastewater purification purposes.^14^ Different parts of the plants have been explored for the synthesis of AgNPs, while some have been synthesized from microbial products (bioflocculants).^1^, ^15^ has reported on silver nano-architecture synthesized from green routes with good potential for decolorization of methylene blue from wastewater. ShirzadiNanotechnology has emerged as a promising approach for cost-effective water treatment solutions, offering high-performance, flexible, highly effective, and multifunctional processes.^16^^,^^17^ The presence of antibiotic-resistant microorganisms in water represents a significant public health concern.^18^ Additionally, the biological approach is primarily employed for monitoring the desired size and shape of nanoparticles, which offers ecological benefits.^19^

Various noble metal nanoparticles, including gold, silver, platinum, and palladium, are extensively utilized in biological research.^3^ Among various types of nanoparticles, silver nanoparticles (AgNPs) have garnered significant attention due to their immense potential for various applications and unique properties.^20^^,^^21^ Undoubtedly, the extensive utilization of this valuable metal in nanoscale form, ranging from household paints to artificial prosthetic devices, has had a profound impact on our daily lives.^22^^,^^23^ The stability, particle size distribution, morphology, and surface/modification all play a crucial role in the controlled synthesis of Ag nanoparticles, generating considerable interest in this area of research.^24^^,^^25^ Studies have demonstrated that silver nanoparticles exhibit inhibitory effects on the growth of fungal and bacterial strains when administered in doses comparable to those used in antibiotic for the treatment of infectious diseases.^26^ Examinations of Ag nanoparticles’ cytotoxicity have uncovered their potential cytotoxic effects on cancerous cells as well as normal cells, including epithelial cells and human lens epithelial cells.^27^ A study by Shirzadi-Ahodashti et al.^28^ has shown AgNPs with good applicability in various fields including wastewater treatment and biomedical field. These Ag nanoparticles were synthesized using Convolvulus *fruticosus* leaf extract. *Streptomyces* sp. MBRC-91 was used to produce a polysaccharide-based bioflocculant on a study by Manivasagan et al..^29^ Their bioflocculant was used to synthesize silver nanoparticles which showed great potential for management of microorganisms on sewage water. Silver nanoparticles were investigated as flocculants in a pilot wastewater treatment plant on a study conducted by Kaegi et al..^30^ The study showed that the synthesized silver nanoparticles were able to trap impurities from both the sludge and effluent.

The present study provides solutions to the limitations of bioflocculants since they are produced in small quantities using high production costs. The synthesis of AgNPs using this approach helps reduce the toxicity of elemental silver which might have a negative impact to the environment and human health. AgNPs and bioflocculants combined provide a synergistic method of treating water. Bioflocculants enhance the physical removal of pollutants by improving the aggregation and sedimentation of suspended particles. AgNPs have potent antibacterial activity and catalytic qualities that enable the effective breakdown of organic contaminants and the removal of pathogens from water.

This study investigates an environmentally friendly method for synthesizing silver nanoparticles using a bioflocculant derived from Kombucha tea SCOBY (Symbiotic Culture of Bacteria and Yeast). The synthesized nanoparticles are evaluated for their efficacy in wastewater treatment, river water purification, and dye removal. Furthermore, cytotoxicity tests on human embryonic kidney (HEK 293) cells, along with antimicrobial activity assays against Gram-positive and Gram-negative bacteria, are conducted to assess the biological and environmental impacts of these nanoparticles. This work demonstrates the integration of bioflocculants and nanotechnology as a sustainable approach to address the global challenges of water pollution and public health.

## 2 Materials and methods

*Chemicals*.

Ethanol (95 % AR grade), Chloroform (AR grade), n-Butyl alcohol (AR grade), and silver nitrate (AgNO_3_, AR grade) were purchased and utilized without any additional purification. Distilled water was used.

### 2.1 Refining and clarifying bioflocculant through extraction and purification

The purification of the bioflocculant was carried out following the methodology outlined by Bakar et al.,^31^ employing a media composition derived from the optimal culture conditions established in earlier experiments. In brief, following 24 h of fermentation, the fermented broth was transferred to centrifuge tubes and subjected to centrifugation at 4000×*g* at a temperature of 4 °C for a duration of 30 min to eliminate bacterial cells. The resulting supernatant was mixed with an equal volume of distilled water and subjected to a subsequent round of centrifugation at 4000×*g*, 4 °C for a duration of 15 min to eliminate any insoluble substances. The supernatant was supplemented with twice its volume of ice-cold ethanol, stirred, and allowed to stand undisturbed at a temperature of 4 °C overnight. The supernatant was discarded, and the resulting precipitate was subjected to vacuum drying to obtain the crude biopolymer. The crude biopolymer was then dissolved in distilled water and mixed with an equal volume of chloroform/n-butyl alcohol mixture (5:2 v/v). Following agitation, the mixture was allowed to stand at ambient temperature for a period of 12 h. The upper phase was isolated, followed by centrifugation at 4000×*g* for 15 min at a temperature of 4 °C. The resulting supernatant was then subjected to overnight dialysis against distilled water. The dialysate was subsequently subjected to vacuum drying to obtain a purified bioflocculant.

### 2.2 Biosynthesis of Ag nanoparticles (AgNPs) and characterization

The synthesis of Ag nanoparticles was conducted following the procedure outlined by Abd Alamer et al.,^32^ incorporating slight adjustments. A 3 mM AgNO_3_ (purchased from Sigma-Aldrich) solution was introduced into a 500 mL flask containing 200 mL of a 10 % bioflocculant solution. The mixture was vigorously stirred for a duration of 2 h and then allowed to sit undisturbed at ambient temperature for a period of 24 h. The precipitate was obtained by subjecting the mixture to centrifugation at 8000×*g* at a temperature of 4 °C for 15 min. The precipitate was subjected to vacuum drying and subsequently pulverized into a fine powder utilizing an agate mortar. The synthesized Ag nanoparticles were visually examined and subsequently subjected to further characterization. The surface morphology and elemental composition of the biologically synthesized Ag nanoparticles were determined using scanning electron microscopy (SEM-EDX) (JOEL USA, Inc., Peabody, Massachusetts 01960, USA) technique. To determine the size and morphology of the samples, transmission electron microscopy (TEM) micrographs were captured using a JEOL 1010 instrument (JEOL, USA) equipped with a Gatan digital camera. The instrument was operated at an accelerating voltage of 100 kV.

The presence of functional groups in the as-synthesized Ag nanoparticles was confirmed by employing Bruker Tensor 27 Fourier transform infrared (FT-IR) spectroscopy (Bruker, Gauteng, South Africa) technique. The XRD pattern samples was recorded utilizing a Bruker AXS D8 advanced diffractometer (Bruker, Johannesburg, SA) equipped with a Ni filter and a CuKꭤ (λ = 1.54056 Å) radiation source. The thermal stability of the samples was assessed using thermogravimetric analysis (TGA) with a TG analyser (PerkinElmer, Inc., Waltham, Massachusetts 02451, USA). The analysis was carried out under a nitrogen atmosphere with a heating rate of 10 °C/min, spanning a temperature range from 22 to 900 °C.

### 2.3 Investigating the impact of Ag nanoparticles dosage concentration on the flocculating activity

Different concentrations of Ag nanoparticles solutions, ranging from 0.2 to 1.0 mg/mL, were prepared by dissolving the Ag nanoparticles powder in distilled water (weight/volume) to achieve the desired concentrations. Afterward, these solutions were utilized to determine the optimal dosage size of Ag nanoparticles. In individual 250 mL conical flasks, the samples (2 mL) were mixed with 100 mL of kaolin solution (0.4 % w/v in distilled water) and supplemented with a 3 mL of 1 % (w/v) CaCl_2_ solution. After vigorous agitation for 1 min, the mixture was transferred into a 100 mL graduated measuring cylinder and allowed to settle for 5 min at ambient temperature. The supernatant was extracted for the assessment of flocculating activity using a spectrophotometer (Pharo 300, Merk KGaA, Germany) at a wavelength of 550 nm.^13^ The flocculating activity (FA) was determined using the following equation.

1 FA(%)=A−B/Ax100

In the equation, A represents the optical density of kaolin solution at 550 nm, and B represents the optical density at 550 nm for the sample.

### 2.4 Antimicrobial activity testing of the synthesized Ag nanoparticles

The selected bacteria for testing were initially revived by inoculating them into sterile nutrient broth and incubating them at 37 °C overnight. Following that, 1 mL of each culture was inoculated into individual test tubes containing 9 mL of sterile nutrient broth. The test tubes were appropriately labelled with the same names of the specific bacteria: *Bacillus cereus, Staphylococcus aureus, Escherichia coli*, and *Pseudomonas aeruginosa*. The culture was subsequently incubated at 37 °C for 24 h. The turbidity of each organism was assessed by measuring the absorbance at 600 nm using a UV–visible spectrophotometer. The turbidity of each strain was subsequently adjusted by adding a fresh sterile nutrient broth to achieve an absorbance within the range of 0.1–0.5, conforming to the accepted standard of McFarland.^33^

#### 2.4.1 Minimum inhibitory concentration (MIC)

The method outlined by Hannan^34^ was employed. The minimum inhibitory concentration (MIC) is defined as the lowest concentration of Ag nanoparticles necessary to inhibit the growth of microorganisms. The quantification of the synthesized nanoparticles was accomplished using 96-well plates. In each well of the 96-well plates, 50 μL of sterile nutrient broth was inoculated. Next, 0.2 g of Ag nanoparticles in 2 mL of distilled water was added. The solution of AgNPs (50 μL) was then added to the first row of the 96-well plates containing nutrient broth and thoroughly mixed. A 3-fold dilution process was conducted by transferring 50 μL from row A to row B of the 96-micro-well plates, followed by thorough mixing. This procedure was repeated with 50 μL taken from row B to the subsequent rows until all the wells contained AgNPs at dissimilar concentrations. The sample (50 μL) in the final column was removed, ensuring that the total volume of all the 96 wells remained at 50 μL. The selected bacterial strains were added (50 L) into their respective wells.

The positive control utilized in this experiment was Ciprofloxacin (40 %), whereas the negative control consisted of distilled water. After the incubation period at 37 °C for 24 h, an indicator solution of *p*-iodonitrotetrazolium violet (INT) was used to assess the results. Thereafter, each well was supplemented with 40 μL of INT solution (0.2 mg/mL) and incubated for additional 30 min at 37 °C. the development of a reddish colour in the wells indicated the metabolic activity of microorganisms, as it signified the reduction of INT to formazan. The absence of a reddish colour (clear) indicated the inactivity of microorganisms, as there was no breakdown of INT to form formazan. The tests were performed in triplicates, and the mean values were recorded.^35^

#### 2.4.2 Minimum bactericidal concentration (MBC)

The minimum bactericidal concentration (MBC) was determined using the agar dilution method. A loopful of the culture from the wells that showed no colour change was streaked onto Muller Hilton nutrient agar. The plates were placed in an incubator at a temperature of 37 °C for 12 h. The minimum concentration of AgNPs at which the test organisms were completely killed was determined as the MBC.^36^

### 2.5 Dye removal efficiency using Ag nanoparticles

Decolorization experiments were conducted by adding 1 mL of Ag nanoparticles into a 50 mL dye solution (4 g/L). The mixture was then shaken for 1 min and allowed to settle for 10 min. The test dyes used in the experiment included safranin, methylene blue, Congo red, and crystal violet, all with a concentration of g/L. After stirring the mixture for 1 min and allowing it to settle for 10 min, the supernatant was collected for analysis using UV-VIS spectrophotometer. The absorbance of each sample was measured at the respective maximum wavelength of each dye, and the decolorization efficiency was calculated using the following equation:

2 $$\text{RE}\;\left(\%\right)=\frac{Co-Cf}{Co}\;x\;100\text{,}$$

Where Co represents the initial value and Cf represents the value after the flocculation treatment. It is crucial to measure the residual concentration of the dye in the samples after treatment, considering the initial and final dye concentrations.^37^

### 2.6 Biosafety assessment of Ag nanoparticles

The biosafety impact of Ag nanoparticles was examined following the method outlined by Singh et al..^38^ The cell lines utilized in this study were the human embryonic kidney 293 (HEK 293) cell lines. The cells were cultivated in 96-well microplates with suspensions at a concentration of 1 × 10^5^ cells/mL. The plates were placed in an incubator and kept at a temperature of 37 °C for 48 h. Following the incubation period, the cells were subjected to the serial dilution method and seeded with dissimilar concentrations of Ag nanoparticles (ranging from 25 to 200 μg/μL). For the administration of nanoparticles, media containing 1 % fetal bovine serum (FBS) were used, and the plates were subsequently re-incubated for an additional 48 h at 37 °C. After incubation period, an indicator in the form of tetrazolium salt (Sigma) was introduced to determine cell viability. Each micro-well was supplemented with approximately 15 μL of MTT (5 mg/mL) prepared in phosphate-buffered saline (PBS), and then incubated at 37 °C for 4 h. The absorbance of the solutions was determined at a wavelength of 570 nm using a microplate reader. The percentage of cell inhibition was calculated using the following equation:

3 Cellviability(%)=Mi/Mfx100,

Where Mi and Mf represent the initial and final values obtained before and after treatment with Ag nanoparticles, respectively.

### 2.7 Application of Ag nanoparticles on pollutants removal from wastewater

Water samples were obtained from Tendele coal mine to evaluate the efficiency of nanoparticles in removing pollutants from the water. When required, the pH value was adjusted using a 1 M NaOH or 1 M HCl. The wastewater sample was subsequently transferred to a 100 mL beaker, and 2 mL of 0.2 mg/mL nanoparticles were added. The mixture was stirred at the specified agitation speed for 10 min and then left undisturbed for 30 min. The upper clear liquid above sediment, was collected for analysis using a spectrophotometer at a wavelength of 680 nm. The levels of residual chemical oxygen demand (COD), biochemical oxygen demand (BOD), total nitrogen (N), calcium (Ca), sulphate (S), and phosphorus (P) were determined in the supernatant. The measurement of total nitrogen, sulphate, COD, BOD, phosphorus, and Calcium was performed using test kits according to the instructions provided by the manufacturer. The removal efficiency (RE) of the pollutants was determined using the sample equation [2].^39^ The results were compared with those of a bioflocculant and commercial flocculant frequently used in wastewater treatment.

## 3 Results and discussion

### 3.1 Characterization of silver nanoparticles

#### 3.1.1 SEM-EDX analysis

**Fig. 1:**
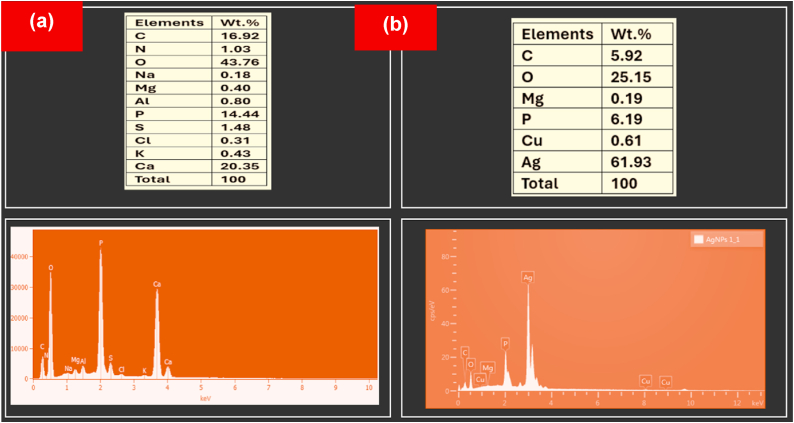
SEM-EDX analysis of (a) bioflocculant and (b) as-prepared Ag nanoparticles.

Fig. 1 illustrates the EDX diagram of a bioflocculant and the silver nanoparticles. The bioflocculant (Fig. 1a) show the presence of elements including C, N, O, Na, Mg, Al, P, S, Cl, K, and Ca. It has been reported elsewhere that the presence of these elements in the bioflocculant is attributed to the elution buffer of the chemical attachment during the column purification.^40^ The presence of Ag nanoparticles (Fig. 1b) was detected at approximately 3 keV in the EDX pattern, which is consistence with the findings of.^41^ Furthermore, a previous investigation conducted by Kgatshe et al.^42^ revealed that absorption peaks below 5 keV indicate the presence of unadulterated silver metal ions. Nonetheless, peaks that corresponds to carbon and oxygen were also noticeable in the EDX spectra, which could be ascribed to the existence of the capping agent derived from the bioflocculant. The EDX spectra indicate that the silver nanoparticles, which were reduced by the bioflocculant contain 61.93 % of silver by weight.

#### 3.1.2 Scanning electron microscopy (SEM) analysis

**Fig. 2:**
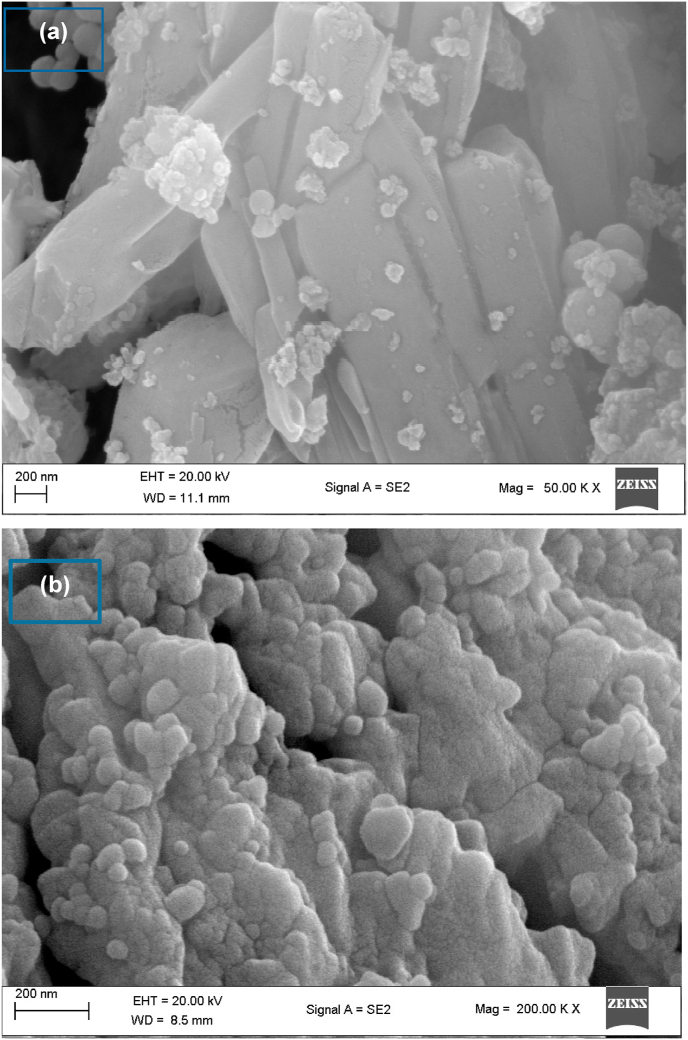
SEM micrographs of (a) bioflocculant and (b) as-prepared Ag nanoparticles.

SEM was implemented to observe the shape size and structural arrangements of silver nanoparticles. Fig. 2 displays the SEM micrographs of the bioflocculant (Fig. 2a) and Ag nanoparticles (Fig. 2b). Observations indicate that the bioflocculant acted as both a reducing and capping agent, resulting in the production of silver nanoparticles with varying shapes. Specifically, spherical, and cuboidal AgNPs were formed due to the reducing and capping properties of the bioflocculant.^43^ The reason for this occurrence could be attributed to the existence of various types and amounts of capping agents within the bioflocculant. Srirangam and Rao^44^ reported SEM micrograph of silver nanoparticles with cuboidal and tubular shapes and size of 30–35 nm. In contrast another study showed that the BsMNPsBF substance exhibited a structure that is agglomerated. Images from the study investigated by Manivasagan et al.^29^ demonstrated the presence of spherical objects thus indicating a distinct morphological property.

#### 3.1.3 FT-IR analysis

**Fig. 3:**
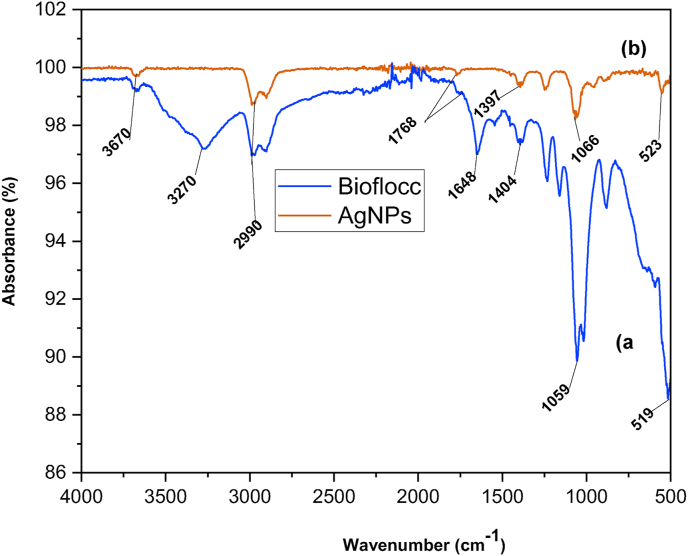
FT-IR spectroscopy analysis of (a) bioflocculant and (b) as-prepared Ag nanoparticles.

FT-IR spectra provided information on the attributes of the surface structure and the functional groups of the bioflocculant that were engaged in reducing Ag ions. The FT-IR spectra of the bioflocculant and silver nanoparticles is displayed in Fig. 3. The bioflocculant's (Fig. 3a) FT-IR spectra exhibited peaks at approximately 3670, 3270, 2990, 1768, 1648, 1059, and 523 cm^−1^. The presence of stretching alcohol can be inferred from the sharp O–H bond, indicated by the peak at 3670 cm^−1^. The presence of deviation peaks at 3670, 2990, 1768, 1066, and 519 cm^−1^ in the as-prepared AgNPs (Fig. 3b) were observed. The stretching hydroxyl (O–H) was assigned to the peak observed at 3670 cm^−1^. The band at 2989 cm^−1^ can be attributed to the C–H stretching vibrations of alkanes, particularly those originating from sp^3^-hybridized carbons.^22^ The presence of the shared vibrational peaks at 3670, 2990, and 1768 cm^−1^ can be observed between the two particles. This provides confirmation that the bioflocculant played a role in the synthesis of AgNPs. These results agree with those reported on a study by Valli et al.^45^ where silver nanoparticles were synthesized using *Cisscus quadrangularis* extracts. The FT-IR data confirms the presence of O–H stretching, which could play a pivotal role in reducing metal ions to their corresponding nanoparticles.^21^ In addition, they stabilize the AgNPs by enclosing them in a protective shell that keeps them from clumping together and guarantees their dispersion.

**Fig. 4:**
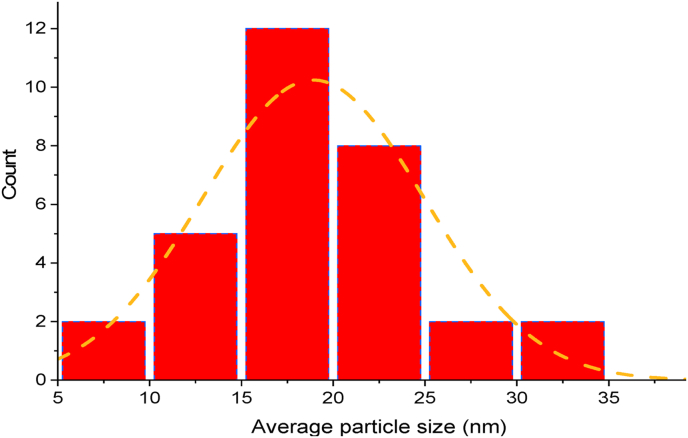
Histogram of Ag nanoparticles showing average particles size distribution.

Fig. S1a (Supplementary Materials) displays the TEM micrograph of the colloidal solution of prepared silver nanoparticles, showcasing a close to spherical shape in a random distribution. The TEM showed silver nanoparticles with average particle size of 18.5 nm. The bioflocculant (Fig. S1a in the Supplementary Materials) revealed particles that were varied in shape, and they appeared spherical and hexagonal with size range between 15.8 and 23 nm. Polydispersed spherical AgNPs were reported with an average particle size of 60 nm.^25^ It has been reported elsewhere that the size and shape of the stabilizing and reducing agents determine the shape and size of the nanoparticles.^46^ Martin et al. utilized *Clitoria ternatea* Linn to synthesize silver-doped ZnO nanoparticles, resulting in spherical particles with sizes ranging from 30 to 40 nm.^47^ Histogram is indicated below (Fig. 4) showing average particle size distribution. From the graph it can be observed that the particles had sizes around 19 nm, this shows that these particles are very small. It has been reported in literature that small particles have high surface area-to-volume ratio which is of great importance during application of these particles in real life situations.^48^

The diffractograms of the purified bioflocculant and the as-prepared silver nanoparticles are depicted in Fig. S2 (Supplementary Materials). The purified bioflocculant exhibited sharp peaks at angles of 2θ = 20.3°, 25.34°, and 32.35°, as observed in the diffractogram. The XRD spectrum of the biosynthesized silver nanoparticles revealed peaks at 2θ = 20.29°, 34.45°, 47.91°, 55.35°, 57.51°, and 72.37°. The observed peaks match the planes labelled as (210), (111), (231), (142), and (311). The mean size of AgNPs obtained in their original state was 19 nm, which closely matches the size determined by TEM analysis, measuring at 18.5 nm. Based on the XRD pattern, Elemike et al.^49^ documented a dominant particle size of 15.8 nm for the leaf-based silver nanoparticles (SNPs). Using X-ray diffraction patterns, the average sizes of PV-AgNPs and SE-AgNPs were found to be 19.32 nm and 15.63 nm, respectively. These results concur to our study findings. Issa et al.^50^ reported on average particles sizes which ranged from 24 to 40 nm as determined by Derby-Scherrer equation. A study by Hashemi et al.^51^ documented the X-ray diffraction analysis with the average sizes of MP-AgNPs and CC-AgNPs to be 34.5 nm and 47.2 nm, respectively. These are contradictory to the study findings but show that different particles can have different sizes and shape depending on the methods used for synthesis. The particles size was estimated using the Derby-Scherrer equation: D = $\frac{K\;\lambda }{\beta \;\mathrm{Cos}\;\theta }$

Fig. S3 (Supplementary Materials) displays the UV–vis spectra of both the purified bioflocculant and the silver nanoparticles. AgNPs were synthesized from a bioflocculant produced by a yeast isolate identified to be *Pichia kudriavzevii* which reduced Ag ^+^ ions from AgNO_3_ to Ag^0^. In Fig. S3a (Supplementary Materials), the purified bioflocculant exhibited a minor absorption band in the range of 250–350 nm, indicating surface plasmon resonance (SPR), with the peak intensity reaching its maximum at 260 nm. In the range of 200–700 nm, a wide range surface plasmon resonance (SPR) band was detected at 410 nm for the AgNPs synthesized. The presence of this band at 410 nm is indicative of the synthesis of AgNPs, as reported by Vanlalveni et al..^52^ A surface plasmon resonance band was reported around 420 nm on a study by Zare-Bidaki et al.^53^ which also showed the successful synthesis of the AgNPs.

Fig. S4 (Supplementary Materials) displays the thermogravimetric analysis (TGA) of the bioflocculant and AgNPs as they undergo heating from 24 to 900 °C. Starting at a temperature of 24–150 °C, degradation of both samples (bioflocculant and AgNPs) commenced, with weight percentages of 3.162 % and 0.212 %, respectively. It has been reported that this degradation is frequently associated with the loss of volatile organic matter and absorbed water. This stage shows that the bioflocculant has been cleared of moisture and low-molecular-weight impurities.^54^ The stability of AgNPs at this phase of degradation suggests that they can function well under ambient or mild heating conditions without suffering a major loss of its functions, which is important for applications like waste management.

Between the temperature of 150 and 200 °C, the as-prepared AgNPs experienced a weight loss of approximately 7.808 wt%, whereas the bioflocculant underwent a more significant weight loss of over 17.212 wt%. The stability of AgNPs is directly related to the integrity of the bioflocculant coating, so, even though the particles are thermally stable far beyond 200 °C. The protective barrier against external factors, including oxidation or contact with other molecules, may be distorted if bioflocculant components are lost during this breakdown. Around the temperature of 250 and 900 °C, the final phase involving the decomposition of residues within the particles, both AgNPs and the bioflocculant experienced weight loss of 6.35 wt% and 17.74 wt%, respectively. From the TGA spectra it can be concluded that the as-prepared AgNPs were thermostable at high temperatures compared to the bioflocculant, the present study findings agree with these results. Kasthuri et al.^55^ reported similar finding from AgNPs synthesized utilizing apiin as a reducing agent.

### 3.2 The effect of dosage size on flocculating activity of Ag nanoparticles

**Fig. 5:**
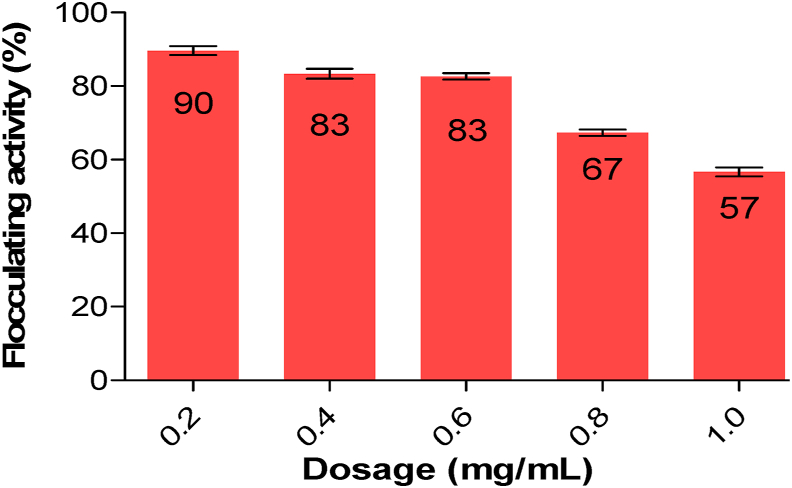
Dosage size effect on flocculating activity of Ag nanoparticles.

The flocculating activity of Ag nanoparticles is depicted in Fig. 5, where a concentration of 0.2 mg/mL exhibited the highest level of flocculating activity, reaching 90 %. In order to identify the ideal concentration of dosage, a range of Ag nanoparticles dosage sizes, varying from 0.2 to 1.0 mg/mL, were examined to assess their flocculating activity. Okaiyeto et al.,^56^ reported that the generation of high viscosity resulting from increased dosage concentrations inhibits the settling rate of solid particles in the solution. In the present investigation, a comparable finding was noted. Fig. 4 illustrates that the highest level of flocculating activity, reaching 90 % at a concentration of 0.2 mg/mL. It was observed that further increases in concentration resulted in a marginal decline in flocculating activity. The marginal reduction in flocculating activity of Ag nanoparticles could potentially be attributed to the elevated viscosity generated by higher concentrations within the solution. This increased viscosity has the tendency to obstruct the adsorption sites for molecules and impede the movement of Ag nanoparticles required for effective flocculation of suspended particles in the solution.^57^

### 3.3 Dye removal

**Fig. 6:**
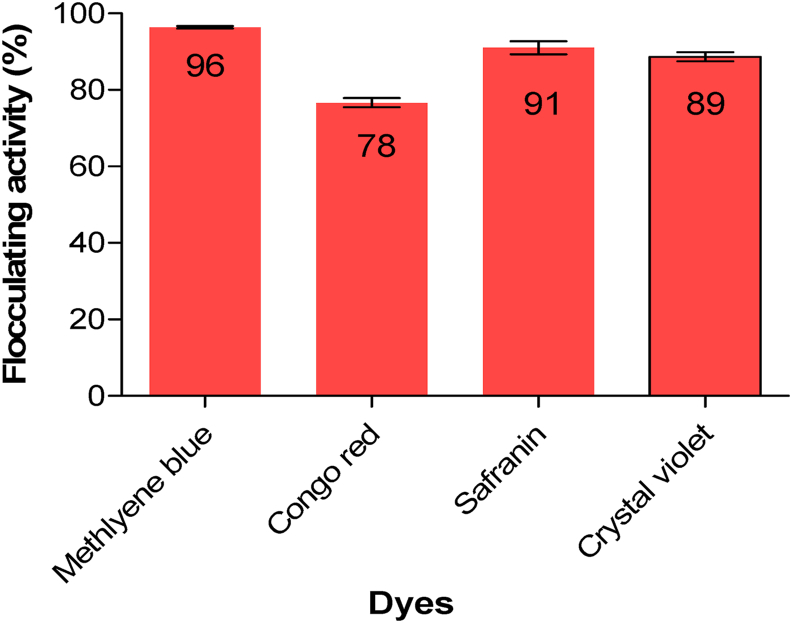
Dye removal efficiency of as-synthesized Ag nanoparticles.

The impact of silver nanoparticles on the removal of staining dyes is presented in Fig. 6. The performance of the Ag nanoparticles in dye removal was satisfactory. The synthesized Ag nanoparticles exhibited a remarkable achievement of up to 96 % removal efficiency for methylene blue and the least removal efficiency of 78 % was observed for safranine. Nevertheless, enhancing the efficacy of Ag nanoparticles may involve increasing the dosage and contact time.^58^ The bioflocculant from which the Ag nanoparticles were synthesized from showed a removal efficiency of above 74 % for safranine, thus indicating a slight increase in removal efficiency of the produced Ag nanoparticles.^59^ Functional groups presented by the FT-IR like carboxyl (-COOH), and amino (-NH_2_) groups are introduced by the bioflocculant coating on AgNPs. These groups interact with the MB dye molecules by van der Waals forces, hydrogen bonds, and electrostatic attraction. This adsorption facilitates further degradation processes by bringing the dye molecules close to the AgNP's reactive surface. The catalytic activity of silver nanoparticles (CH-AgNPs), produced by trisodium citrate solution (Na_3_C_6_H_5_O_7_), for the degradation of both orange and blue dye alone and in combination was investigated by Kaushik et al..^60^ Following the experiment, NaBH_4_ shows around 30 % and 25 % removal efficacy for blue and orange dyes, respectively, as the dye concentration in solution increases from 50 ppm to 200 ppm. However, when CH-AgNPs + NaBH_4_ were used, the blue dye showed a 100 % decline, whereas the orange dye showed about 98 % degradation. These findings concur with those reported in the present study highlighting the efficiency of AgNPs in dye degradation.

### 3.4 Antimicrobial activity test

Table 1 illustrates the relative effectiveness of Ag nanoparticles compared to Ciprofloxacin (Commercial antibiotic) in terms of their antimicrobial activity. The antimicrobial activity test was conducted to assess the impact of the biologically synthesize Ag nanoparticles on both Gram-positive Gram-negative bacterial strains. The Gram-positive bacterial strains (*B. cereus* and *S. aureus*) showed to be less susceptible to the synthesized Ag nanoparticles with concentrations 6.25 and 12.5 mg/mL, respectively. While they were more susceptible to Ciprofloxacin with concentrations 1.56 and 6.25 mg/mL, and the bioflocculant did not show any positive results for any of the tested strains. The Gram-negative (*P. aeruginosa* and *E. coli*) on the other hand were more susceptible to Ag nanoparticles at concentrations 3.125 mg/mL. This could be due to that Ag nanoparticles can penetrate the outer membrane of Gram-negatives more easily because of their small size as shown by the TEM results and interact directly with the cell membrane, leading to cell damage and death.^61^ Additionally, Gram-negative bacteria have a higher affinity for Ag ions due to their thinner peptidoglycan layer, which allows easier penetration and interaction with intracellular targets.^62^ The AgNPs’ antibacterial efficacy against these pathogenic microbes is increased by the functional groups including but not limited to carboxyl and polyphenols, which help stabilize the particles and improve their contact with bacterial cells, reactive oxygen species production, and silver ion release. Sathiyanarayanan et al.,^63^ reported Ag nanoparticles that were synthesized using a polysaccharide bioflocculant produced from a marine *Bacillus subtilis* MSBN17, which showed a high antimicrobial activity against Gram-negative bacteria (*P. aeruginosa*). Another study has reported AgNPs with a good antimicrobial activity against *S. aureus* and *E. coli* with 0.1 μg/mL sensitivity for both microorganisms.^4^ Table 2 below compares the antibacterial of the present study with those reported in literature and it is well comparable with good results.

**Table 1:** MIC and MBC of Ag nanoparticles in comparison to Ciprofloxacin and the bioflocculant.

Bacteria	AgNPs		Ciprofloxacin		Bioflocculant	
	MIC (mg/mL)	MBC (mg/mL)	MIC (mg/mL)	MBC (mg/mL)	MIC (mg/mL)	MBC (mg/mL)
*Bacillus cereus*	6.25	–	1.56	3.125	–	–
*Staphylococcus aureus*	12.5	–	6.25.	12.5	–	–
*Pseudomonas aeruginosa*	3.125	6.25	6.25	6.25	–	–
*Escherichia coli*	3.125	6.25	3.125	6.25	–	–

**Table 2:** Antibacterial activity comparison with existing literature.

Study	Nanoparticle type	Synthesis method	Bacteria tested	Key findings	Novelty
Current study	Bioflocculant-based AgNPs	Kombucha SCOBY	*S. aureus and E. Coli*	High antibacterial activity	Synergistic actions mediated by functional groups that utilize polyphenols, carboxyl groups, and Ag^+^ release
Hadi et al.^64^	Plant-derived AgNPs	*Diplazium esculentum* extract	*E. coli and S. aureus*	Moderate production of ROS and concentration-dependent bactericidal actions.	In this study, they produced a AgNPs with improved stability and focused effect.
Vu et al.^65^	Chemically synthesized AgNPs	Sodium borohydride reduction	*S. aureus and E.Coli*	High toxicity but significant environmental impact due to residual chemicals.	Environmentally friendly synthesis without toxic chemical residues.

### 3.5 Cytotoxicity assay

**Fig. 7:**
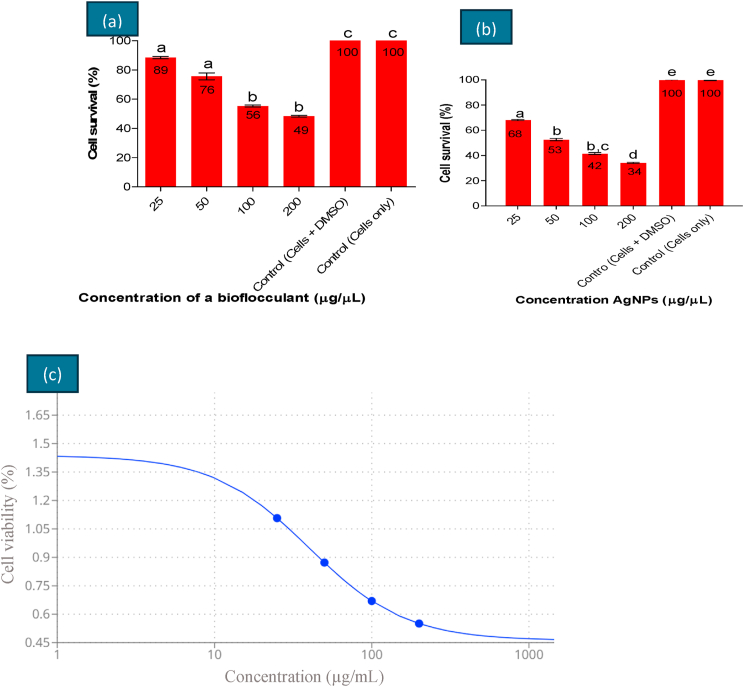
Cytotoxicity assay of (a) bioflocculant (b) Ag nanoparticles on Human embryonic kidney (HEK 293) cells and (c) the IC50 value.

The standard methyl thiazolyl tetrazolium (MTT) assay was employed using human embryonic kidney (HEK 293) cells to assess the cytotoxicity of the bioflocculant (Fig. 7a) and Ag nanoparticles (Fig. 7b). The HEK 293 cell line is commonly used as a toxicity model to study the potential effects of human exposure to Ag nanoparticles.^66^ The cell survivability was reduced to 88.5 % for the bioflocculant at a concentration of 25 μg/μL while that of the synthesized Ag nanoparticles dropped significantly to 68 %. With an increase in concentration (from 25 to 200 gμ/μL) the synthesized Ag nanoparticles were posing a high effect on the viability of the cells (HEK 293). At the highest concentration of Ag nanoparticles (200 μg/μL) the cell viability was 34 % while that of the bioflocculant was 49 %, thus indicating that the synthesized Ag nanoparticles have increased cytotoxicity effect on human embryonic kidney (HEK 293) cell line. The IC50 value of this assay was measured at 40.04 μg/mL. The IC50 value for AgNP–S was determined to be 117.43 μg/mL, while the IC50 values for CNL, CNS, AgNP-L, and C–AgNP exceeded 200 μg/mL, indicating lower cytotoxicity for these samples on a study reported by Chiu et al..^67^ At the maximum tested concentration of 200 μg/mL, cell viability was higher for the plant extracts, with CNL at 77.05 % and CNS at 72.10 %. In contrast, the synthesized nanoparticles exhibited greater cytotoxicity, with AgNP-L showing 57.24 % viability and AgNP–S displaying the lowest viability at 26.47 %. While the AgNP–S in the literature shows slightly lower viability at 200 μg/mL (26.47 %) compared to the present study with 34 %, our findings are still consistent in demonstrating significant cytotoxicity at high concentrations.

Literature has reported that Ag nanoparticles may induce apoptosis or necrosis on HEK 293 cell line and generate Reactive Oxygen Species (ROS) which induces the oxidative stress in the cells.^66^ The cytotoxic effects of the synthesized Ag nanoparticles (AgNPs) on HEK 293 cells can be explained by their ability to induce oxidative stress, a widely reported mechanism of nanoparticle toxicity^68^ Upon cellular uptake, AgNPs interact with cellular components, leading to the generation of ROS. The elevated levels of ROS can overwhelm the cell's natural antioxidant defenses, resulting in oxidative stress. This stress disrupts normal cellular functions by damaging lipids, proteins, and DNA, which ultimately compromises cell viability.

Similar findings were reported where a dose-dependent cytotoxicity effect of Ag nanoparticles was observed on a study by Sooklert et al..^69^ The DCFH-DA assay demonstrated that a 24-h exposure to SS-AgNPs (*Semenovia suffruticosa* silver nanoparticles) at concentrations of 25, 50, 100, and 200 μg/mL resulted in a significant elevation of reactive oxygen species (ROS) production in cells. This finding suggested that SS-AgNPs induce oxidative stress in the cells. Another investigation has documented a similar trend in cell viability with respect to concentrations. They have reported a concentration dependent activity were an increase in the amount of AgNPs resulted in the decrease in cell survivability. The IC50 value of the AgNPs was determined to be 200 μg/mL which agrees with our study findings.^53^

### 3.6 Treatment of wastewater using Ag nanoparticles

The results in Table 2 demonstrate that Ag nanoparticles exhibited effective removal capabilities for various pollutants including biological oxygen demand (BOD), chemical oxygen demand (COD), phosphorus, sulphate, total nitrogen, and calcium in coal mine wastewater. The synthesized Ag nanoparticles revealed noteworthy efficacy in the removal of these elements. The findings indicate that Ag nanoparticles have the potential to serve as a viable substitute for chemical flocculants. The passivated Ag nanoparticles with bioflocculant coatings offer properties, such as degradability and environmental friendliness, which are absent in chemical flocculants.^70^ The presence of elevated levels of BOD and COD in water is detrimental to aquatic life and these pollutants should be removed.^71^ High concentrations of P, S, N, and Ca in water stimulates the process of eutrophication.^72^ Among the tested flocculants, the synthesized Ag nanoparticles showed the highest removal efficiencies for BOD and COD, with removal rates of 92 % and 86 %, respectively. These results of the Ag nanoparticles were better compared to the microbial and iron (III) chloride flocculants as these showed removal efficiencies of 80 % and 76 %, 90 % and 65 % for BOD and COD, respectively. The effective removal of the Ag nanoparticles can be attributed to their surface structure, chemical composition, and functional groups. The nanoparticles' surface is enriched with functional groups including carboxyl, alcohols, polyphenols, etc., from the bioflocculant used in their synthesis. These groups enhance interactions with negatively charged pollutants, such as organic matter, and ionic contaminants, thus promoting flocculation process as observed. Furthermore, van der Waals forces might also be involved in the binding of organic molecules that are neutral.^73^

The synthesized Ag nanoparticles demonstrated effective removal of P, S, N, and Ca in Tendele coalmine wastewater with removal efficiencies of 86, 92, 75, and 92 %, respectively. The removal efficiencies for the microbial flocculant for the pollutants P, S, N, and Ca were 81, 87, 54, and 90 %, respectively. While the removal efficiencies of the commercial flocculant (FeCl_3_) for pollutants P, S, N, and Ca were 69, 93, 57, and 70 %, respectively. Chen et al.^74^ documented AgNPs with removal efficiency of 99 % for phosphorus. It can, therefore, be concluded that the as-synthesized Ag nanoparticles can be used to remove pollutants from industrial wastewater since it showed better results compared to tested commercial flocculant (FeCl_3_) frequently used in wastewater treatment industries. The flocculating activity was also conducted at a wavelength of 680 nm, and it was observed (Table 3) that Ag nanoparticles show a good flocculating activity of 97 % compare to the bioflocculant and iron (III) chloride.

**Table 3:** Removal efficiency of Ag nanoparticles for pollutants in coal mine wastewater in comparison to FeCl_3_ and microbial base flocculant microbial flocculant.

Flocculants	Water quality	Pollutants						Flocculation activity @680 nm
		BOD	COD	P	S	N	Ca	
Microbial	Before treatment	212 ± 0.1	150 ± 0.0	21 ± 0.3	150 ± 0.2	14 ± 0.1	115 ± 0.0	1.529
	After treatment	42.1 ± 0.2	36 ± 0.2	4 ± 0.1	19 ± 0.0	6.5 ± 0.4	12 ± 0.1	0.241
	**Removal rate**	**80**	**76**	**81**	**87**	**54**	**90**	**84**
AgNPs	Before treatment	212 ± 0.1	150 ± 0.0	21 ± 0.0	150 ± 0.2	14 ± 0.1	115 ± 0.0	1.529
	After treatment	18 ± 0.1	21 ± 0.3	3 ± 1.0	12 ± 0.2	3.5 ± 0.3	9 ± 1.0	0.037
	**Removal rate**	**92**	**86**	**86**	**92**	**75**	**92**	**97**
FeCl_3_	Before treatment	212 ± 0.1	150 ± 0.0	21±	150 ± 0.2	14 ± 0.1	115 ± 0.0	1.529
	After treatment	20.7 ± 0.0	23 ± 0.2	6.5±	10.2 ± 0.0	6 ± 0.0	35 ± 0.3	0.136
	**Removal rate**	**90**	**65**	**69**	**93**	**57**	**70**	**91**

## 4 Conclusion

This study successfully demonstrated the bioflocculant-mediated synthesis of silver nanoparticles (AgNPs), with comprehensive characterization using SEM–EDX, TEM, UV–Visible spectroscopy, FT-IR, XRD, and TGA. The biosynthesized AgNPs exhibited remarkable antimicrobial properties, effectively inhibiting the growth of both Gram-negative and Gram-positive pathogenic bacteria. Furthermore, the AgNPs displayed concentration-dependent cytotoxic effects on HEK 293 cell line, with cell viability decreasing from 68 % at 25 μg/μL to 34 % at 200 μg/μL. In comparison to commercial flocculants such as iron (III) chloride, the biosynthesized AgNPs demonstrated superior performance in pollutant removal. When applied to coalmine wastewater, they achieved removal efficiencies exceeding 91 % for biological oxygen demand (BOD) and 85 % for chemical oxygen demand (COD) at a concentration of 0.2 mg/mL. Additionally, the AgNPs showed significant dye removal capabilities, achieving an overall efficiency of over 78 %, with a maximum of 96 % for methylene blue.

These findings highlight the potential of biosynthesized AgNPs as an eco-friendly and efficient alternative to synthetic flocculants for wastewater treatment and environmental remediation. Their multifunctional applications in pollutant removal and antimicrobial activity present a promising avenue for addressing water pollution challenges sustainably in the future.

## CRediT authorship contribution statement

**Phakamani H. Tsilo:** Writing – review & editing, Writing – original draft, Investigation, Formal analysis. **Albertus K. Basson:** Supervision, Conceptualization. **Zuzingcebo G. Ntombela:** Writing – review & editing, Supervision, Methodology. **Nkosinathi G. Dlamini:** Writing – review & editing, Supervision, Methodology. **Rajasekhar V.S.R. Pullabhotla:** Writing – review & editing, Supervision, Methodology, Funding acquisition, Formal analysis, Conceptualization.

## Funding

Rajasekhar Pullabhotla would like to acknowledge the 10.13039/501100001321National Research Foundation (NRF, South Arica) for their financial support in the form of the Incentive Fund Grant (Grant no. 103691) and the Research Developmental Grant for Rated Researchers (112,145). Phakamani Tsilo would like to acknowledge the Council for Scientific and Industrial Research (CSIR, South Africa) for the financial assistance in the form of the Doctoral bursary.

## Declaration of competing interest

The authors declare the following financial interests (e.g., any funding for the research project)/personal relationships (e.g., the author is an employee of a profitable company) which may be considered as potential competing interests:Rajasekhar Pullabhotla would like to acknowledge the National Research Foundation (NRF, South Arica) for their financial support in the form of the Incentive Fund Grant (Grant no. 103691) and the Research Developmental Grant for Rated Researchers (112,145).
