# Supplementary material for: Applications of silver nanoparticles synthesized from Pichia kudriavzevii bioflocculant isolated from Kombucha tea SCOBY
Source: Biotechnol Notes. 2025 Feb 21;6:106–16. doi: 10.1016/j.biotno.2025.02.003 (PMC11930702; doi:10.1016/j.biotno.2025.02.003)
Supplement: Multimedia component 1 [file mmc1.docx]

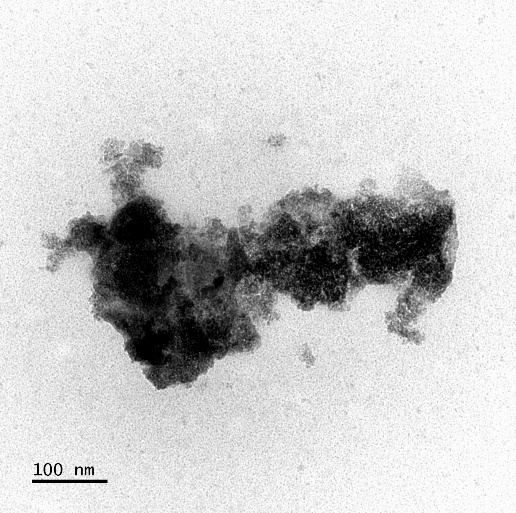

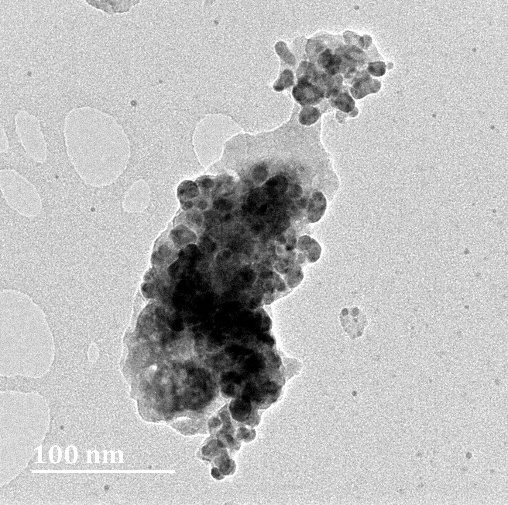


**(b)**

**(a)**

**Figure S1.** TEM images of the (a) bioflocculant and (b) as-prepared AgNPs.

**Figure S2.** X-ray diffraction of bioflocculant and as-prepared AgNPs.

**(a)**

**(b)**

**Figure S3.** UV-vis spectra of the (a) bioflocculant and (b) as-prepared AgNPs.

**Figure S4.** Thermogravimetric spectra of the bioflocculant and as-prepared AgNPs.
